# Cardiac Arrest-Induced Global Brain Hypoxia-Ischemia during Development Affects Spontaneous Activity Organization in Rat Sensory and Motor Thalamocortical Circuits during Adulthood

**DOI:** 10.3389/fncir.2016.00068

**Published:** 2016-08-25

**Authors:** Michael Shoykhet, Jason W. Middleton

**Affiliations:** ^1^Department of Pediatrics, Washington University School of Medicine in St. LouisSt. Louis, MO, USA; ^2^Department of Pediatrics, St. Louis Children’s HospitalSt. Louis, MO, USA; ^3^Department of Cell Biology and Anatomy, School of Medicine, Louisiana State University Health Sciences CenterNew Orleans, LA, USA; ^4^Neuroscience Center of Excellence, School of Medicine, Louisiana State University Health Sciences CenterNew Orleans, LA, USA

**Keywords:** spontaneous activity, correlation, ischemic injury, sensory thresholds, developmental period

## Abstract

Normal maturation of sensory information processing in the cortex requires patterned synaptic activity during developmentally regulated critical periods. During early development, spontaneous synaptic activity establishes required patterns of synaptic input, and during later development it influences patterns of sensory experience-dependent neuronal firing. Thalamocortical neurons occupy a critical position in regulating the flow of patterned sensory information from the periphery to the cortex. Abnormal thalamocortical inputs may permanently affect the organization and function of cortical neuronal circuits, especially if they occur during a critical developmental window. We examined the effect of cardiac arrest (CA)-associated global brain hypoxia-ischemia in developing rats on spontaneous and evoked firing of somatosensory thalamocortical neurons and on large-scale correlations in the motor thalamocortical circuit. The mean spontaneous and sensory-evoked firing rate activity and variability were higher in CA injured rats. Furthermore, spontaneous and sensory-evoked activity and variability were correlated in uninjured rats, but not correlated in neurons from CA rats. Abnormal activity patterns of ventroposterior medial nucleus (VPm) neurons persisted into adulthood. Additionally, we found that neurons in the entopeduncular nucleus (EPN) in the basal ganglia had lower firing rates yet had higher variability and higher levels of burst firing after injury. Correlated levels of power in local field potentials (LFPs) between the EPN and the motor cortex (MCx) were also disrupted by injury. Our findings indicate that hypoxic-ischemic injury during development leads to abnormal spontaneous and sensory stimulus-evoked input patterns from thalamus to cortex. Abnormal thalamic inputs likely permanently and detrimentally affect the organization of cortical circuitry and processing of sensory information. Hypoxic-ischemic injury also leads to abnormal single neuron and population level activity in the basal ganglia that may contribute to motor dysfunction after injury. Combination of deficits in sensory and motor thalamocortical circuit function may negatively impact sensorimotor integration in CA survivors. Modulation of abnormal activity patterns post-injury may represent a novel therapeutic target to improve neurologic function in survivors.

## Introduction

Acute brain injury (e.g., hypoxia-ischemia due to stroke or cardiac arrest [CA]) disrupts sensory processing, motor function, cognition and behavior in survivors (Volpe et al., 1984; Graves et al., 1997; Schreckinger et al., 2007). Injury-induced alterations include inflammation, astrocyte and microglial activation, excitotoxicity, axonal and dendritic remodeling and changes in intrinsic and synaptic neural function (Wong et al., 1993; Barone and Feuerstein, 1999; Werner and Engelhard, 2007; Winship and Murphy, 2008, 2009). While injury-induced structural and functional changes will impact sensory and motor function and sensorimotor integration, the developmental time point at which injury occurs may independently affect functional maturation of neural circuitry. Spontaneous and evoked activities are both critical for normal development; therefore, injury affecting both will disrupt normal development. In order to develop better therapeutic and rehabilitative interventions, we must understand how neural injury during development affects maturation and functional organization of neuronal circuits in adulthood.

Evidence that ongoing spontaneous activity during development exerts a lasting effect on the organization and function of neuronal circuits in adulthood has emerged from both sensory and motor systems. Before the onset of visual experience, retinal ganglion cells fire spontaneous bursts of action potentials that contribute to coherent propagating waves of activity across the retina (Wong et al., 1993). These waves can engage burst-dependent Hebbian synaptic plasticity rules to refine retinogeniculate synapses (Butts et al., 2007). Ongoing spontaneous activity in the brain can predict a large degree of stimulus response variability (Arieli et al., 1996; Petersen et al., 2003) and can act to gate cortical responses to sensory stimuli (Luczak et al., 2013). Spontaneous correlations in inhibitory and excitatory neural activity in the cortex mediate stimulus-response decorrelation via a disynaptic feedforward inhibitory circuit (Middleton et al., 2012). Spontaneous sequences of activity contain signatures of sensory evoked responses (Han et al., 2008; Luczak et al., 2009; Berkes et al., 2011; Eagleman and Dragoi, 2012; Luczak and MacLean, 2012) and sensory activity can stabilize neural assemblies through reinforcement of patterned synaptic connectivity (Litwin-Kumar and Doiron, 2014). In the motor system, corticospinal axons in the spinal cord remodel their arbors in response to changes in activity of primary sensory afferents (Jiang et al., 2016). In the developing motor cortex (MCx), spontaneous gamma and spindle bursts correlate with motor and sensory inputs associated with movement (An et al., 2014).

In this study, we used two previously collected data sets from rats that underwent asphyxial CA during development to explore further the impact of injury on the relationship between spontaneous and evoked activity in the somatosensory system (Shoykhet et al., 2012) and on the variability and firing modes in the thalamic entopeduncular nucleus (EPN) in the motor system (Aravamuthan and Shoykhet, 2015). The EPN is located slightly ventral to the ventrobasal complex containing ventroposterior medial nucleus (VPm) and represents a rodent homolog of human and monkey Globus Pallidus pars interna (GPi). EPN, like GPi, sends output from the basal ganglia to the motor thalamus and the MCx. Abnormal activity patterns in GPi and other basal ganglia nuclei are associated with movement abnormalities in Parkinson’s disease and in dystonia. In the somatosensory thalamus, spontaneous activity of thalamocortical neurons correlated with specific aspects of their evoked responses in sham but not in injured rats. In the EPN, CA-induced hypoxia-ischemia followed by months of recovery was still associated with higher variability of spontaneous firing and increased frequency of bursting. Altogether, the findings from our analysis highlight that key features of spontaneous activity organization in sensory and motor brain areas remain persistently altered months after global brain hypoxic-ischemic injury. These alterations may significantly impact development, organization and function of thalamocortical circuit that underlie sensory processing, sensorimotor integration, cognition and behavior.

## Materials and Methods

The analyses in this manuscript utilize data acquired during two previously published studies (Shoykhet et al., 2012; Aravamuthan and Shoykhet, 2015). The experimental procedures are briefly described below.

### Animals

All animal procedures were approved by the Institutional Animal Care and Use Committees at the University of Pittsburgh and at Washington University in St. Louis. For recordings in the VPm, male Sprague-Dawley rats were housed with their mother until postnatal day 30 (P30). Food and water were provided *ad libitum*. A total of 32 rats were used in this study. For neurophysiologic experiments, the rats underwent either CA (*n* = 7) or sham (*n* = 9) intervention at P17–19 (see below, CA and resuscitation in “Materials and Methods” Section). After CA, the rats were further divided into early and late groups. Electrophysiological experiments were performed on these groups 2–3 days and 6–8 weeks, respectively, after injury or sham treatment. The early group consisted of six sham and three injured rats, the late group of three sham and four injured rats. The experimenter was blind to the injury status of the rats at the time of the recordings and data analyses. There were no grossly observable differences in appearance or behavior between injured and sham rats in either the early or the late groups. Specifically, all rats in the study groomed, whisked, ate and drank. There were no differences in posture or gross locomotion in the cage, and no obvious differences in anesthetic requirement or physiologic parameters during the recordings. For recordings in the EPN and the MCx, adult Long-Evans rats of both sexes were used. There were eight rats each in the CA and sham groups in this experiment. CA and sham procedures at P17–19 were identical to those used in VPm recordings. Rats were allowed to mature until 6–8 months of age prior to EPN/MCx recordings.

### Cardiac Arrest and Resuscitation

The model of developmental asphyxial CA has been described in detail previously (Fink et al., 2004). Briefly, P17–19 rats underwent tracheal intubation and placement of femoral artery and venous lines under isoflurane/nitrous oxide anesthesia. The rats were mechanically ventilated under neuromuscular blockade. Arterial blood pressure, electroencephalogram and electrocardiogram were continuously monitored and recorded. Core body temperature was maintained constant at 37°C with a servo-controlled heating blanket. The anesthetic was then briefly washed out with room air, and rats were disconnected from the ventilator. Asystole ensued within 60–90 s of apnea and was allowed to continue for 9 min in rats used for VPm recordings and 9.5 min for rats used in EPN/MCx recordings. The rats were then resuscitated with mechanical ventilation using 100% oxygen, intravenous epinephrine, sodium bicarbonate and manual chest compressions. Upon return of spontaneous circulation, the rats additionally received a 20 ml/kg bolus of 5% dextrose in normal saline intravenously to prevent dehydration. After ~2–3 h, mechanical ventilation was discontinued, the rats were extubated, arterial and venous lines were removed and wound margins were sutured. All wound margins were infiltrated with lidocaine. The rats were observed for an additional 1 h in a chamber with 100% oxygen to mimic a clinical scenario and then returned to the mother.

### Surgical Preparation for Electrophysiological Recordings

Surgical procedures in developing animals have been described previously (Shetty et al., 2003; Shoykhet et al., 2003; Shoykhet and Simons, 2008). Either 2–3 days (early) or at least 6 weeks (late) after asphyxial CA, rats were anesthetized with isoflurane, and underwent: (1) tracheotomy; (2) placement of an external jugular venous and femoral arterial catheters; and (3) craniotomy. A steel post was affixed to the skull with dental acrylic to allow holding the rat’s head without pressure points for the remainder of the experiment. The craniotomy (~2 mm^2^) was performed through the skull overlying the appropriate region of interest. Dura mater was left intact. A ground screw was placed through the skull and fixed with dental acrylic. After completions of all surgical procedures, the rat was transferred to the vibration isolation table and placed in a custom-made head holder. Mechanical ventilation using a volume-controlled Inspira ventilator (Harvard Apparatus) was initiated using tidal volumes of ~8 ml/kg and rates of 100 and 70 breaths/min in younger and older rats, respectively. Neuromuscular blockade was initiated with a bolus dose of pancuronium bromide (~1 mg/kg) and maintained with a continuous infusion of pancuronium (0.8 mg · kg^−1^ · h^−1^) in 5% dextrose/0.9% sodium chloride for the remainder of the experiment. Isoflurane anesthesia was then discontinued, and the rat was transitioned to fentanyl analgesia using continuous fentanyl infusion at ~8–10 μg · kg^−1^ · h^−1^. At these doses, the rats enter a state of dissociative analgesia without compromise of thalamocortical network dynamics observed under anesthesia (Simons et al., 1992).

The rat’s physiologic state during the recording session was continuously monitored as described previously (Shoykhet and Simons, 2008). Briefly, temperature was maintained at 37°C using a servo-controlled heating blanket (Harvard Apparatus) and, in young rats, a 20W DC lamp. Intra-arterial pressure and heart rate (HR) were monitored using a blood pressure monitor (WPI Inc.) connected to the arterial line via a pressure transducer. If mean arterial pressure (MAP) did not remain in the developmentally appropriate range, experiments were discontinued. Adequate analgesia was assured throughout the recording session by monitoring pupillary constriction and by maintaining a lack of MAP and HR elevation in response to gentle touch. At the end of the recording session, the rats were deeply anesthetized with 5% isoflurane in room air and perfused transcardially for cytochrome oxidase (CO) histochemistry. Recording electrode tracks were visible in thionin or Nissl stained brain slices (Aravamuthan and Shoykhet, 2015).

### Electrophysiological Recordings

Extracellular recordings were obtained using stainless steel microelectrodes (6–8 ΩM impedance at 1 kHz; FHC, Bowdoinham, ME, USA). The electrode was advanced perpendicularly to the pia using a hydraulic micropositioner (David Kopf Instruments, Tujunga, CA, USA). Entry into VPm was signaled by an abrupt increase in spontaneous and evoked neural activity both in sham and in injured animals. In both groups, VPm was somatotopically organized, individual units were easily isolated and there were no silent zones. Signals were initially bandpass filtered between 300 Hz and 10 KHz and amplified 10- to 100-fold using a DC-powered preamplifier (Grass Instruments). The resulting signal was further filtered using a 60 Hz notch and a bandpass filter (BAK Electronics) and digitized using a PCI-MIO-16E4 data acquisition board (National Instruments) connected to a personal computer.

For EPN and MCx recordings, the rats were transferred from the surgical table to a stereotaxic frame (David Kopf Instruments). Electrodes in MCx were positioned at a 20° angle to pial surface through a craniotomy (1.5 mm rostral to bregma and 3.0 mm lateral to midline) and manually advanced to a depth of 1 mm with a fine 3-axis micropositioner (David Kopf Instruments) to target cortical Layer V. Electrodes for EPN recordings were advanced perpendicularly to pial surface through a craniotomy (2.3 mm caudal to bregma and 3.0 mm lateral to midline) with a hydraulic microdrive (David Kopf Instruments). EPN location was determined via its position directly ventral to VPm and ventral posterolateral nucleus (VPl). After traversing VPm and VPl, relative quiescence indicated passage through the internal capsule, followed by an abrupt increase in activity indicating entry into EPN. For both MCx and EPN recordings, the raw wide-band signal was passed through a 1× headstage, digitized at 40 kHz/channel, and digitally filtered between 1 and 300 Hz for local field potential (LFP) recordings and between 300 Hz and 10 kHz for spike trains (Plexon Inc., Dallas, TX, USA). Continuous wide band, LFP and spike train signals were stored in .plx format for off-line analyses.

### Whisker Stimulation and Data Acquisition

Facial vibrissae were deflected using a multi-angle piezoelectric stimulator (Simons, 1983) controlled by custom-written LabView software. The stimulator was attached to the whisker 5 mm from the face in young rats and ~10 mm from the face in adults. The stimuli consisted of a hold-ramp-hold pattern, delivered over a total of 500 ms. The stimulator was calibrated to deflect the whisker at ~125 mm/s; the deflection amplitude was 0.5 mm in young rats and 1 mm in adults, resulting in equivalent angular deflection (~5.7°) in all age groups. The whisker was deflected in eight standard directions, with deflection in each direction repeated 10 times for a total of 80 stimuli per whisker. Deflections were interleaved in a random manner. All sensory-evoked responses in this study were calculated by averaging individual trial responses to all eight whisker deflection angles, while the study that previously presented the thalamocortical unit data analyzed only responses to the best angle of deflection (Shoykhet et al., 2012). The 25 ms value of the counting window during the deflection onset (and offset) was chosen to cover the transient increase and return to near baseline levels after onset, while excluding an excessive number of plateau (or spontaneous) spikes that would dilute the true response values. The plateau counting window of 125 ms was chosen because it covers a period of the sustained plateau response in relative equilibrium after recovery from the onset increase in activity.

Data were collected for 500 ms; action potential time stamps were collected with 100 μs resolutions. Action potential waveforms were digitized at 32 kHz and stored for off-line analyses. Off-line, the action potential waveforms were sorted using custom written software that allowed for isolation of single-unit recordings based on a combination of waveform parameters, including principal component analysis, deflection slopes and amplitude of peaks and valleys. To confirm further the single-unit nature of the recordings, sorted spikes were examined using an interspike interval (ISI) histogram; only recordings with <1% of the sorted spikes within the absolute refractory period (1 ms) were used in the analyses.

For simultaneous EPN and MCx recordings, 5–10 min of spontaneous activity for each EPN unit were recorded, and 300 s of data free from artifacts was used for analyses. Single units were sorted using Plexon Offline Sorter (Plexon Inc.) based on visualization of 3D clusters in principal component space. All isolated units had an absolute refractory period >1 ms. Since all neurons in EPN are GABA-ergic (Kha et al., 2000), we did not analyze individual waveforms to differentiate between excitatory and inhibitory cells (Simons, 1978; Bruno and Simons, 2002).

### Statistical Treatment of the Data

Alpha-trimmed means were used to compare average values among populations. An α-trimmed mean is defined as the sample mean derived from the set of observations *n* from which κ largest and smallest values have been removed, and where κ is the next largest integer of α × *n* (Fisher and van Belle, 1993). α-trimmed means are more robust with respect to the effect of outliers, and statistical analyses using α-trimmed means become more conservative due to a reduction in the degrees of freedom and inclusion of only the most frequently observed values in the calculations. For statistical comparison of mean values between groups that are defined by injury (sham or CA) and time point (early or late) we used a two-way ANOVA followed by multiple pairwise comparisons using Tukey’s honest significant difference (HSD) test (“anova2” and “multcompare” in Matlab). Unless otherwise stated, *indicates *p* < 0.05, **indicates *p* < 0.01 and ***indicates *p* < 0.001. The correlation was calculated using Spearman’s correlation coefficient. Spearman’s correlation is the Pearson’s correlation coefficient of the indexed ranks of two data sets. This measure is robust to the influence of statistical outliers. Power spectra were calculated using Welch’s method of power spectral estimation and the mean-square coherence (“pwelch” in Matlab). The pairwise comparison of Sham vs. CA data in the EPN single unit recordings was performed using either a student *t*-test or Wilcoxon Rank Sum test after normality of the data was determined using the Liliefors test for normality.

## Results

### Magnitude and Variability of Whisker Somatosensory Thalamic Neuron Spontaneous and Sensory-Evoked Activity

**Figure 1:**
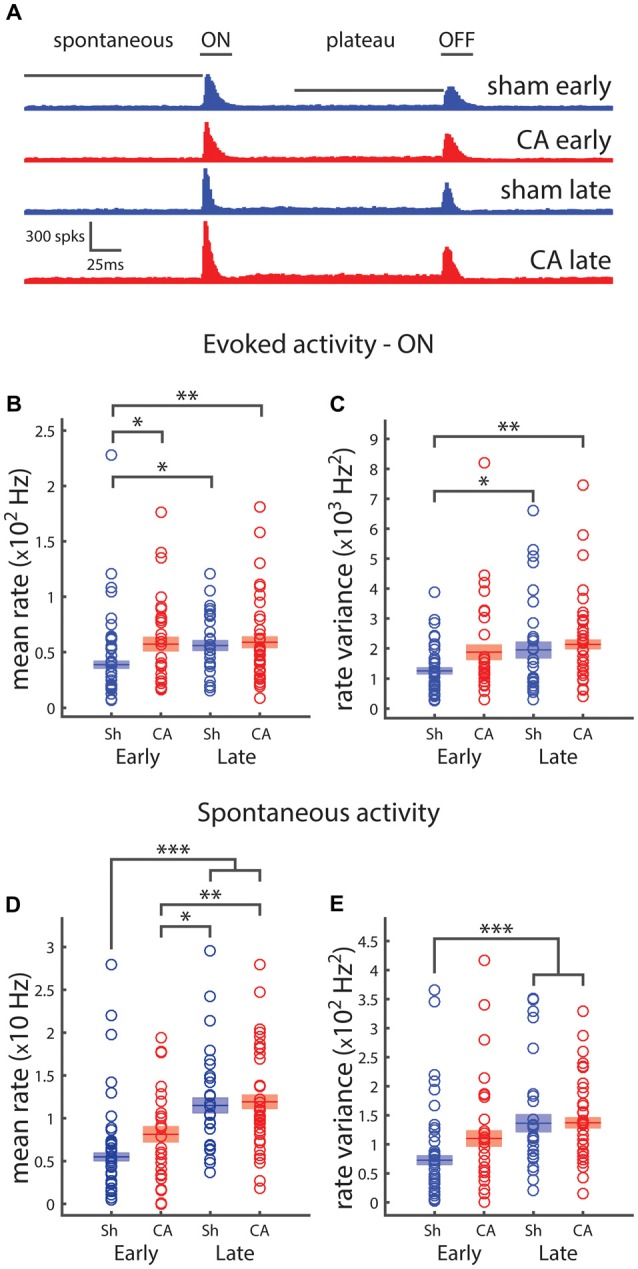
**Spiking activity of thalamocortical neurons depends on development and is affected by ischemic injury. (A)** The poststimulus time histogram (PSTH) for whisker ramp and hold deflection stimuli responses of sham (blue) and cardiac arrest (CA; red) neurons during early and late periods. The horizontal bars indicate the time periods over which spikes are counted for response counts in spontaneous, ON, plateau and OFF periods. **(B)** Average firing rates for evoked whisker deflection (onset: ON) response. Early sham responses are significantly smaller than all other group averages. **(C)** Average firing rate variance of whisker responses reveal that early sham responses are less variable than both late responses. **(D)** Firing rates during the 150 ms spontaneous period preceding deflection. Early sham and early CA firing rates are smaller than both late groups. **(E)** Firing rate variance of whisker responses reveals that early sham spontaneous activity is less variable that both late groups. Horizontal bars and rectangular shaded regions in **(B–E)** indicate mean ± S.E.M. of the α-trimmed data sets. *Indicates *p* < 0.05, **indicates *p* < 0.01 and ***indicates *p* < 0.001.

We used extracellular recordings of action potentials of thalamic barreloid (VPm) neurons and quantified spontaneous activity and responses to whisker deflection sensory stimuli. Poststimulus time histograms show rapid increases in neural firing in response to whisker deflection onsets and offsets in recordings from four groups of rats: sham-early, CA-early, sham-late and CA late (Figure 1A). We quantified whisker deflection-evoked responses by calculating the evoked spike rates averaged in the 25 ms period after whisker deflection. A two-way ANOVA was conducted to understand the effects of ischemic injury and age on response and variance. We found that the effect of both injury (*F*_(1,109)_ = 5.13, *p* = 0.024) and age (*F*_(1,109)_ = 4.0, *p* = 0.046) were significant (Figure 1B). *Post hoc* pairwise comparisons using Tukey’s HSD test revealed significant differences between evoked firing rates of sham-early neurons (38.9 ± 3.2 Hz) and CA-early neurons (57.2 ± 6.1 Hz, *p* = 0.031), as well as between sham-late neurons (55.9 ± 4.5 Hz, *p* = 0.048) and CA-late neurons (58.9 ± 4.8 Hz, *p* = 0.008; Figure 1B). The firing rate variability of whisker-evoked responses was quantified by calculating the trial-to-trial rate variance in the 25 ms post-stimulus period. There were significant effects of both injury (*F*_(1,109)_ = 4.71, *p* = 0.032) and age (*F*_(1,109)_ = 6.65, *p* = 0.011) on variance (Figure 1C). *Post hoc* comparisons revealed significant pairwise differences between sham-early neuronal firing rate variance (13.2 ± 1.2 × 10^2^ Hz^2^) and sham-late (21.9 ± 3.5 × 10^2^ Hz^2^) and CA-late (24.1 ± 2.7 × 10^2^ Hz^2^) firing rate variances (Figure 1C). The neural activity of VPm neurons during spontaneous conditions were quantified by calculating the average spike firing rate in the 150 ms period before whisker deflection. The effects of both injury and age were significant: *F*_(1,109)_ = 4.22, *p* = 0.042 and *F*_(1,109)_ = 43.4, *p* = 1.6e^−9^, respectively (Figure 1D). There were significant pairwise differences between sham-early spontaneous rates (5.49 ± 0.45 Hz) and sham-late (11.5 ± 0.9 Hz, *p* = 4e^−7^) and CA-late (11.9 ± 0.8 Hz, *p* = 1e^−8^) firing rate variances. Additionally, there were significant pairwise differences between CA-early (8.13 ± 0.89 Hz) and sham-late (*p* = 0.017) and CA-late (*p* = 0.003) spontaneous firing rates (Figure 1D). We only observed a main effect of age on spontaneous firing rate variances: *F*_(1,109)_ = 17.4, *p* = 6e^−5^ (Figure 1E). Pairwise significance differences were observed between sham-early (7.26 ± 0.76 × 10^1^ Hz^2^) and sham-late (14.5 ± 1.7 × 10^1^ Hz^2^, *p* = 3e^−4^) and CA-late (15.2 ± 1.1 × 10^1^ Hz^2^, *p* = 1e^−4^) firing rate variances (Figure 1E).

**Figure 2:**
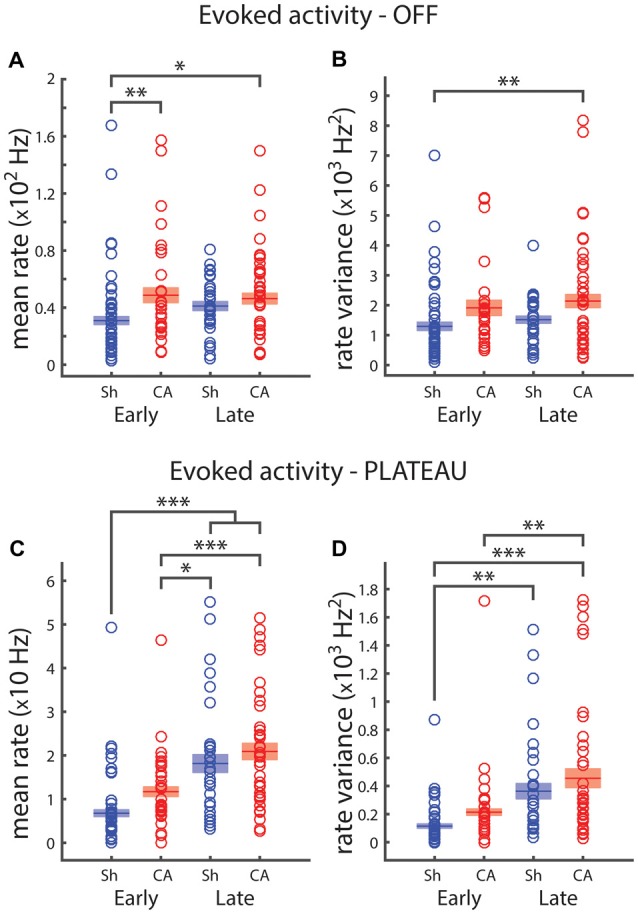
**Plateau and OFF responses depend on development and are affected by ischemic injury. (A)** Early sham average OFF response rate is significantly lower that both CA groups. **(B)** Firing rate variance of OFF whisker responses reveals that early sham response is less variable that late CA response. **(C)** Firing rates for the 125 ms plateau period preceding whisker offset. Early sham rate is smaller than both late group rates; early CA rate is smaller than both late group rates. **(D)** Early sham plateau responses are less variable than both late group responses; early CA firing rate variance is smaller than late CA firing rate variance. Horizontal bars and rectangular shaded regions indicate mean ± S.E.M. of the α-trimmed data sets. *Indicates *p* < 0.05, **indicates *p* < 0.01 and ***indicates *p* < 0.001.

The response of VPm neurons to ramp and hold stimuli exhibits an elevated plateau of activity during the sustained whisker deflection and a transient response to the deflection offset, when the stimulator returns the whisker to its resting position (OFF responses, Simons and Carvell, 1989). We examined how OFF and plateau responses depend on exposure to brain hypoxia-ischemia and on the developmental period. Two-way ANOVA revealed a significant effect of injury on OFF whisker-deflection response firing rates: *F*_(1,109)_ = 9.5, *p* = 0.003 (Figure 2A). Pairwise *post hoc* comparison revealed significant differences between sham-early OFF firing rates (30.9 ± 2.8 Hz) and CA-early (48.7 ± 5.1 Hz, *p* = 0.006) and CA-late (46.3 ± 3.7 Hz, *p* = 0.012) offset response firing rates (Figure 2A). Similarly, there was a significant effect of injury on OFF firing response variance: *F*_(1,109)_ = 10.6, *p* = 0.002 (Figure 2B). We observed pairwise significant differences between sham-early (13.9 ± 1.5 × 10^2^ Hz^2^) and CA-late (23.6 ± 3.0 × 102 Hz^2^, *p* = 0.006) OFF response rate variance (Figure 2B). When we examined whisker plateau activity (in between whisker deflection onset and offset, Figure 1A), we found that both injury and age had an effect on plateau firing rates (Figure 2C). *Post hoc* pairwise comparisons revealed significant differences between sham-early (6.8 ± 0.8 Hz) and sham-late (18.1 ± 2.0 Hz, *p* = 3e^−6^) and CA-late (20.9 ± 1.9 Hz, *p* = 5e^−9^) firing rate responses. There were also significant difference responses between CA-early responses (11.6 ± 1.1 Hz) and sham-late (*p* = 0.029) and CA-late (*p* = 3e^−4^) firing rates.

Both injury and age had an effect on plateau firing rate variance: *F*_(1,109)_ = 4.31, *p* = 0.04 and *F*_(1,109)_ = 28.2, *p* = 6e^−7^, respectively. There were significant pairwise differences between sham-early (12.3 ± 1.8 × 10^1^ Hz^2^) and sham-late (41.1 ± 7.1 × 10^1^ Hz^2^, *p* = 0.001) and CA-late (48.6 ± 8.0 × 10^1^ Hz^2^, *p* = 1e^−6^) plateau firing rate variances. There was also a significant difference between CA-early (21.7 ± 2.4 × 10^1^ Hz^2^) and CA-late (*p* = 0.002) plateau rate variance (Figure 2D).

**Figure 3:**
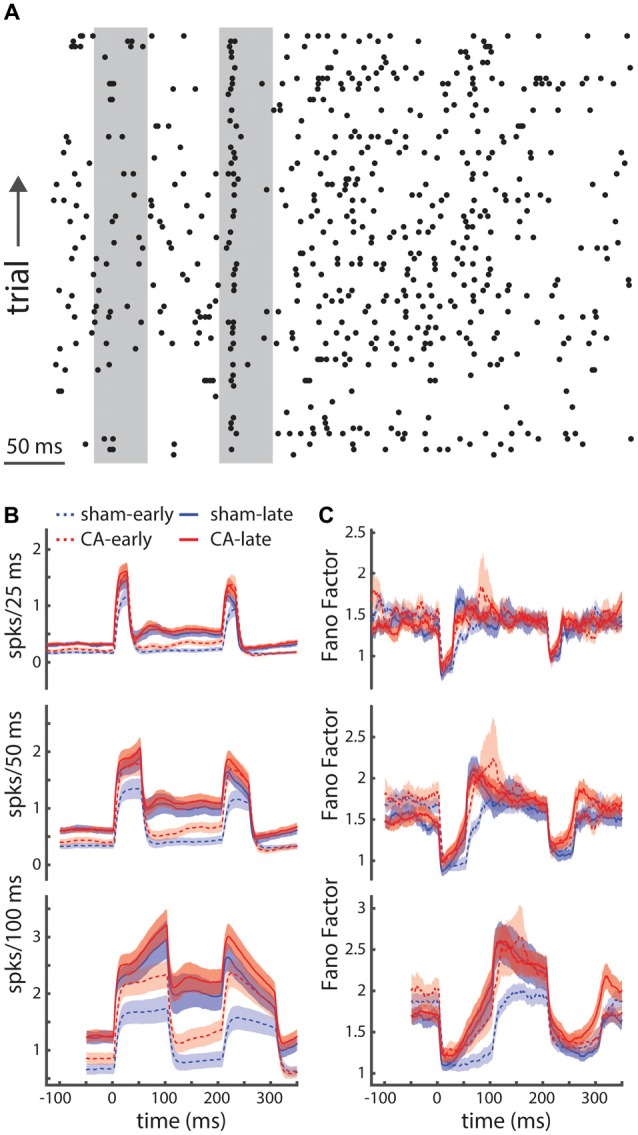
**Relative spike-count variability is unique for early sham rats. (A)** A spike raster plot displaying the relatively high trial-to-trial variability of thalamocortical neurons in the ventral posterior medial (VPm) nucleus. **(B)** Time dependent firing rates are calculated by convolving spike counts with sliding square-wave windows of different widths (25 ms, 50 ms and 100 ms; top-bottom). **(C)** The time-dependent Fano factor (spike count variance divided by mean count), using different counting windows (top-bottom: 25 ms, 50 ms, 100 ms), reveals stimulus dependent reduction in spike count variability. Using a large counting window (100 ms) we are better able to estimate the Fano factor and start to see systematic differences in the pattern of relative variability of sham-early spiking activity compared to other groups: both CA-early and late groups. Shaded regions indicate s.e.m.

We can see that both the amplitude and relative variability of spike counts are different between spontaneous periods and stimulus-evoked periods (Figure 3A: left and right gray bars, respectively). Our approach to examining the state-dependent nature of the spike count variability involved using a sliding counting window. We examined spike counts using sliding counting windows of different widths (Figure 3B). The sliding nature of the window allows us to examine variations in mean spike counts that may change on timescales smaller than the window itself. However, counts in time points that are closer than the counting window width will be correlated. We used these time-dependent spike count profiles and their corresponding spike count variances to estimate the Fano-factor. The Fano factor of a point process is the ratio of the count variance to mean count in a given window. We show Fano factor as a function of time for different count windows (Figure 3C). As observed in other sensory neurons, we see that the Fano factor decreases with whisker deflection onset. Fano factors increase with the counting window width (top-bottom) and the sensory dependent reduction becomes more apparent with large counting windows (e.g., 100 ms shown in the bottom panel). What also becomes apparent is the unique time course of the stimulus-evoked reduction in the Fano-factor from sham-early neurons. While the Fano factor for CA-early, sham-late and CA-late neurons initiates a return to baseline shortly after the stimulus-evoked minimum, the Fano-factor for sham early neurons remains low for a time period on the order of ~50 ms. As the counting windows used to reveal this different behavior is large (100 ms) this span of persistently low Fano factor includes both whisker deflection evoked spikes and early plateau spikes. This result implies that the neural activity of sham-early neurons may exhibit a unique form of stimulus-dependent reduction of neural variability that is absent in CA-early neurons. This contrast may have differential consequences on how thalamocortical activity engages cortical circuits during important developmental periods.

### Correlation of Average Spontaneous and Sensory-Evoked Activity

**Figure 4:**
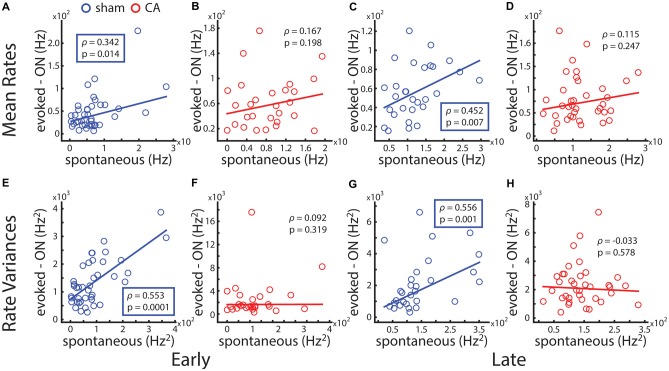
**Correlation of spontaneous and deflection-evoked activity is affected by ischemic injury. (A)** The average level of spontaneous activity a thalamocortical neuron exhibits and its average whisker evoked stimulus response count is positively correlated across the population of neural recordings in early sham rats. **(B)** Spontaneous-evoked rate correlation is not significant for early CA rats. **(C)** Spontaneous-evoked rate correlation is positive and significant for late sham rats. **(D)** Spontaneous-evoked rate correlation is not significant for late CA rats. **(E)** Spontaneous-evoked variance correlation is positive and significant for early sham rats. **(F)** Spontaneous-evoked variance correlation is not significant for early CA rats. **(G)** Spontaneous-evoked variance correlation is positive and significant for late sham rats. **(H)** Spontaneous-evoked variance correlation is not significant for late CA rats.

To understand the functional connection between spontaneous activity and sensory activity in the whisker somatosensory thalamus, we calculated the correlation coefficient between observed spontaneous spike counts and whisker-evoked spike counts. The mean spontaneous rate and evoked rate across a population of sham-early VPm neurons is positively correlated (ρ = 0.342, *p* = 0.028; Figure 4A), while spontaneous and evoked rates are uncorrelated for CA-early neurons (ρ = 0.167, *p* = 0.394; Figure 4B). A similar spontaneous-evoked correlation is observed for late animals: spontaneous and evoked spike rates are correlated for sham-late VPm neurons (ρ = 0.452, *p* = 0.013; Figure 4C) and uncorrelated for CA-late neurons (ρ = 0.115, *p* = 0.494, Figure 4D). Firing rate variance and mean firing rates observed from neural recordings in the brain generally exhibit a positive increasing relationship (Goris et al., 2014). To confirm that the correlation of spontaneous and whisker-evoked firing rate variance exhibits the same dependence as on mean firing, we additionally calculated the correlation coefficient between these measures from our data sets. The spontaneous and evoked firing rate variances of sham-early VPm neurons are positively correlated (ρ = 0.553, *p* = 2e^−4^; Figure 4E) and uncorrelated for CA-early neurons (ρ = 0.092, *p* = 0.638; Figure 4F). A similar spontaneous-evoked correlation structure is observed for late animals: spontaneous and evoked firing rates are correlated for sham-late VPm neurons (ρ = 0.556, *p* = 0.002; Figure 4G) and uncorrelated for CA-late neurons (ρ = −0.033, *p* = 0.845, Figure 4H).

### Timing of Whisker Somatosensory Thalamic Neuron Sensory-Evoked Activity

**Figure 5:**
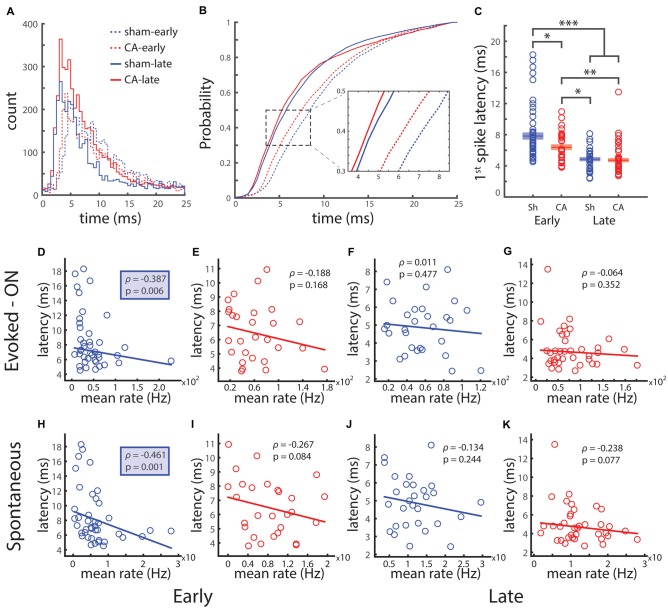
**Spike timing of whisker-evoked responses depends on development and is affected by ischemic injury. (A)** PSTHs for the 25 ms whisker deflection ON period. **(B)** The cumulative distribution of spike times during the 25 ms ON period. Zoom: the population average time to 40% of spikes shows that the same proportion of spikes occurs earlier for late groups compared to early groups and for CA-early compared to sham-early. **(C)** The latency between whisker deflection onset and the first spike in trials with non-zero response is significantly longer for sham-early vs. all other groups, and CA-early vs. late groups. **(D)** The first spike latency and evoked spike count is negatively correlated for the early sham group. **(E–G)** First spike latency and evoked count are uncorrelated for CA-early and both late groups. **(H)** First spike latency and spontaneous spike count are negatively correlated for sham-early rats. **(I–K)** First spike latency and spontaneous spike count are not significantly correlated for early CA and both late groups. *Indicates *p* < 0.05, **indicates *p* < 0.01 and ***indicates *p* < 0.001.

The peri-stimulus time histograms (PSTHs) of whisker evoked responses (Figure 5A) and their corresponding cumulative probability distributions (Figure 5B) reveal an ordered relationship of response timing after whisker deflection. The inset shows that the average time it takes to observe 40% of the spikes in the counting window is smaller in both late populations, followed by CA-early neurons and then sham-early neurons. To quantify the relative distributions of early stimulus responses, we calculated the average first spike latencies in the 25-ms counting windows. Note that this measure differs from the response latency as calculated in a prior study (Shoykhet et al., 2012). The response onset latency in that study detects the first stimulus-dependent response that is statistically distinguishable from spontaneous activity, while the first spike latency we calculate is more dependent on the temporal location of the highest slope of the PSTH. We performed two-way ANOVA to understand the influence of injury and age on first spike latency. There were significant effect of both injury and age on latency: *F*_(1,109)_ = 5.94, *p* = 0.016 and *F*_(1,109)_ = 52.5, *p* = 7e^−10^, respectively. The interaction effect of injury and age on first spike latency was significant: *F*_(1,109)_ = 4.18, *p* = 0.043. *Post hoc* pairwise comparison of latencies revealed significant differences between sham-early (7.8 ± 0.4 ms) and CA-early (6.4 ± 0.3 ms, *p* = 0.010), sham-late (4.9 ± 0.2 ms, *p* = 1e^−8^) and CA-late (4.7 ± 0.2 ms, *p* = 4e^−9^) first spike latencies (Figure 5C). There were also significant differences between CA-early and sham-late (*p* = 0.010) and CA-late (*p* = 0.002) latencies.

We calculated the correlation of first spike latency and stimulus evoked spike count to see if there was a relationship between early stimulus timing and stimulus magnitude. The first-spike time and spike count for sham-early neurons were negatively correlated (ρ = −0.387, *p* = 0.012), while they were uncorrelated for CA-early (ρ = −0.188, *p* = 0.335), sham-late (ρ = 0.011, *p* = 0.953) and CA-late (ρ = −0.064, *p* = 0.703) neurons (Figures 5D–G). We saw that the magnitude of the sensory-evoked responses was correlated with the average spontaneous activity of that neuron (Figure 4). To see if the average first-spike latency of a neuron was also related to its level of spontaneous activity, we calculated the correlation coefficient of these two quantities. The first spike-latency and average spontaneous spike count for sham-early neurons were negatively correlated (ρ = −0.461, *p* = 0.002) while they were uncorrelated for CA-early (ρ = −0.267, *p* = 0.168), sham-late (ρ = −0.314, *p* = 0.488) and CA-late (ρ = −0.238, *p* = 0.154) neurons (Figures 5H–K). This unique relationship between initial response timing and both spontaneous and sensory evoked activity in early healthy thalamocortical neurons may play an important role in the transmission of thalamocortical activity during critical periods.

### Activity in the Motor Thalamus and Motor Cortex

**Figure 6:**
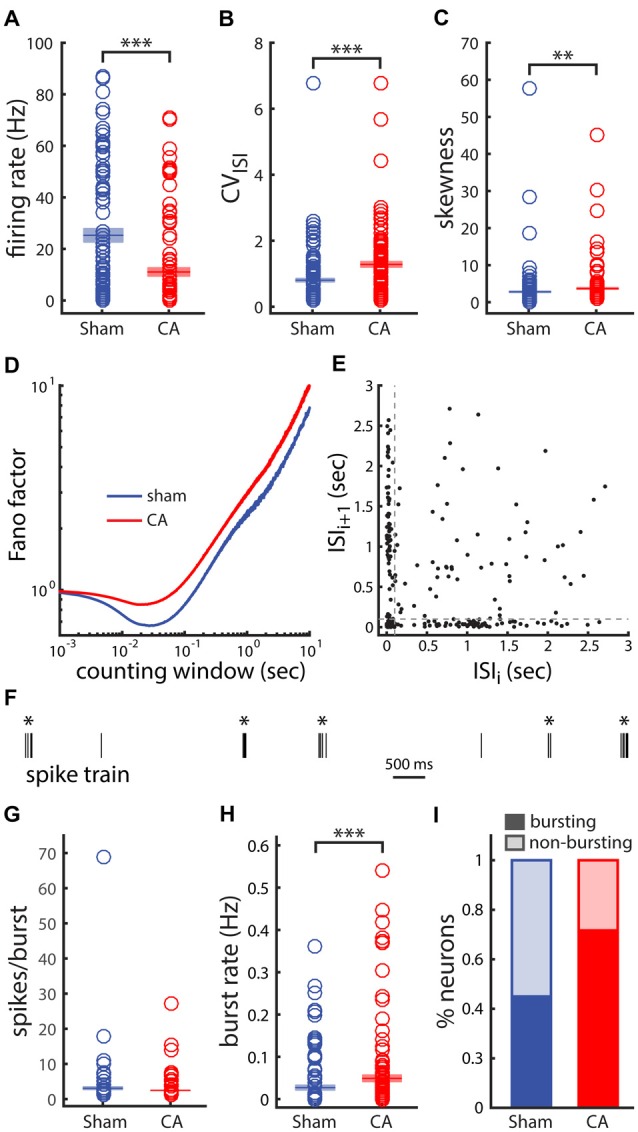
**Variability and burst firing of activity from neurons in the entopeduncular nucleus (EPN) of the basal ganglia is affected by ischemic injury. (A)** Firing rate is lower in CA neurons than in Sham neurons as previously observed. **(B)** The coefficient of variation (CV) of the interspike intervals (ISIs) is larger in CA neurons than in Sham neurons.** (C)** The skewness (a measure of asymmetry) of the ISI histogram (ISIH) of CA neurons is larger than in Sham neurons. This asymmetry arises from a relatively larger, more distributed tail of ISI values in the CA neuron ISIHs.** (D)** The Fano-factor of spike counts as a function of counting window is larger for Sham neurons than CA neurons for the range of counting windows examined.** (E)** The ISI return map for an example CA neuron reveals burst-like firing in this case. The spread of points along the axes arise from a long ISI followed by a short ISI and vice versa. These ISI pair sequence arise at the initiating of a burst and at the end of a burst, respectively.** (F)** The spike train of the same example neuron shows clearly distinguishable burst and isolated spike events. **(G)** The number of spikes per burst in Sham and CA neurons is similar. **(H)** The burst rate averaged across all neurons in the groups is larger for CA neurons than for Sham neurons. **(I)** 45% of Sham neurons exhibit burst activity compared to 72% of CA neurons. *Indicates *p* < 0.05, **indicates *p* < 0.01 and ***indicates *p* < 0.001.

We also examined whether increased spontaneous firing rates of thalamic neurons represent a general feature of circuit reorganization following hypoxia-ischemia. As in the original study (Aravamuthan and Shoykhet, 2015), we observed decreased firing rates of EPN neurons in CA rats compared to sham rats (Figure 6A). However, variability in ISIs, measured by the coefficient of variation (CV = SD/mean), and skewness of the ISI histogram (ISIH; 3rd moment/SD^3^) both increased in CA rats compared to sham rats (Figures 6B,C). The CV is a measure of spike train variability—low CVs (>1) reflect temporally regular spike train firing patterns and a Poisson spike train has a CV of 1 (Cox, 1962; Nawrot et al., 2008). CVs >1 can arise from larger membrane fluctuations (Middleton et al., 2003; Ackman et al., 2012; Siegel et al., 2012), fluctuating inputs and variable spike generating mechanisms (Churchland et al., 2006; Failor et al., 2010) or bursting spike activity (Tripp and Eliasmith, 2007; Failor et al., 2010). The Fano factor of spike counts as a function of the counting window length is also higher at all time scales in CA compared to sham neurons (Figure 6D), further indicating increased relative spike count variability with injury. As the counting window approaches ~0 both Fano factors converge to 1, the Fano factor of a Poisson process (Cox, 1962; Simons and Land, 1987; Shoykhet et al., 2005) and both curves have relative minima near the inverse of the mean firing rate (Carvell and Simons, 1996; Middleton et al., 2003). To further examine the ISI variability and confirm that the increase observed in CA neurons is related to burst firing, we examined more closely the ISI sequences from spike trains in each group. ISI return maps (scatter plot of ISIs vs. the ISIs immediately preceding them in sequence) can reveal temporal correlations and non-randomness in ISI sequences (Avila-Akerberg and Chacron, 2011). An example ISI return map from a CA spike train in our data set illustrates non-random correlation features consistent with burst firing. The elongated clusters near the axes correspond to sequential ISI pairs transitioning from long to short intervals during the commencement of a burst (or vice versa for the end of a burst), and the cluster near the origin illustrates sequential short intervals that occur within bursts (Figure 6E). For our dataset we used a lower bound threshold of 100 ms to determine inclusion of shorter intervals in bursts. Additionally, we set a higher threshold of 250 ms that set the requirement that intervals preceding and following a sequence of short intervals needed to be longer than this threshold in order for the sequence to be defined as a burst. A sample segment of the spike train from the same neuron shows the efficacy of these classification criteria to separate visibly distinguishable burst events (asterisks) from single isolated spike events (Figure 6F). We found that on average the number of spikes counted in each observed burst was not distinguishable between sham and CA neurons (sham: 4.10 ± 0.37, CA: 3.49 ± 0.23, *p* = 0.106; Figure 6G). However, when averaged across all neurons the rate of burst events firing (bursts/second) in sham neurons (0.027 ± 0.005 Hz) was lower than observed for CA neurons (0.049 ± 0.007 Hz, *p* = 6.8e^−4^; Figure 6H). The proportion of Sham neurons that displayed burst firing was 0.45 (36/80) while the proportion of CA neurons that displayed burst firing was 0.722 (52/72; Figure 6I). A Chi-Squared test for equality of binomial proportions confirmed that the prevalence of burst firing differed significantly between sham and CA neurons in the EPN (*χ*^2^ = 11.5, *p* = 0.0007). These data indicate that the firing rates of EPN neurons, unlike those of VPm neurons, decrease in CA survivors, and suggest that post-injury functional abnormalities in the thalamus may be circuit-specific. Decreased firing rates in EPN after CA are associated with an increased spiking variability that is related to a change in burst firing prevalence.

**Figure 7:**
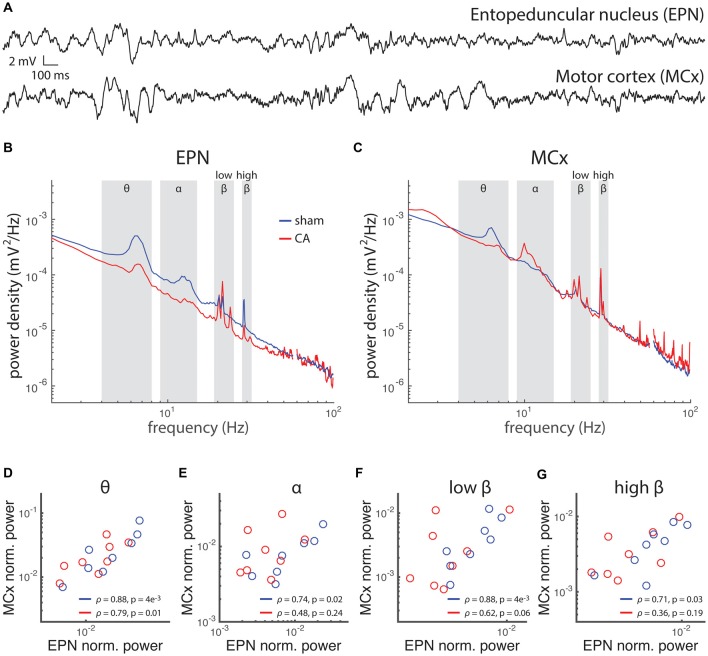
**Rhythmic activity and correlation between motor cortex (MCx) and basal ganglia is affected by hypoxic ischemic injury. (A)** Local field potentials (LFPs) were simultaneously recorded in the EPN of the basal ganglia and the MCx in rats 6 weeks after injury or sham treatment. **(B)** The power spectral density of EPN LFPs in sham rats and CA rats exhibit spectral peaks at several frequencies, implying relatively more pronounced rhythms contributing to the LFP fluctuations at those frequencies. The frequency bandwidths are labeled: θ (4–8 Hz), α (9–15 Hz), low-β (19–25 Hz) and high-β (28–32 Hz). **(C)** The power spectral density of MCx LFPs exhibits relative peaks in the same frequency bandwidths of EPN LFPs. **(D–G)** Power in each bandwidth was averaged and normalized to the mean power across all frequencies. Average power in MCx was plotted against average power in EPN for each rat. The correlation between MCx and EPN mean power levels in different bandwidth was examined across the rats in our study by calculating the Spearman correlation coefficient of these two area-specific powers. We found that Sham rats had correlated MCx-EPN LFP power levels in the θ, α and low-β ranges, while CA rats had correlated MCx-EPN LFP power levels in the θ band only.

Abnormalities of long-range circuit functional connectivity after CA are also evident in simultaneous LFP recordings in EPN and MCx (Figures 7A–C). We previously reported that coherence between EPN and MCx LFP is decreased in CA survivors. Here, we extend this finding further by demonstrating that the normalized total power of EPN LFP covaries with that of MCx LFP across multiple frequency bands in sham rats (Figures 7D–G). We did not observe such covariation in CA rats, however, except in the low frequency Θ range. Each data point represents a single average value from LFPs in multiple recording electrode placements in the same animal. Breakdown of covariance between EPN and MCx LFP’s in CA rats occurs despite the fact that the overall LFP power remains similar between CA and sham rats in both brain locations. Abnormal brain rhythms and coherence of rhythms between different areas have been implicated in a number of neurological diseases (Babiloni et al., 2004; Sarnthein and Jeanmonod, 2007; Schulman et al., 2011; Ping et al., 2013). The relative independence of power levels in different bandwidths of LFP fluctuations between EPN and MCx may underlie some form of functional decoupling between these areas during development, which is supported by the previous finding that the temporal coherence between these areas is also reduced with ischemic-hypoxia induced injury (Aravamuthan and Shoykhet, 2015). This differential long-range organization may arise from the way the different levels of burst activity in EPN that we observed engages postsynaptic plasticity mechanisms in MCx.

## Discussion

Normal organization and function of information processing in the cortex relies on sensory-dependent experience, but equally importantly relies on network patterns of spontaneous activity during pre- and peri-critical developmental periods (Katz, 1993; Wong, 1993; Wong et al., 1995). Spontaneous thalamocortical activity is essential for engaging synaptic mechanisms that established the refined topical organization in cortical circuits (Crair and Malenka, 1995; Ackman et al., 2012; Siegel et al., 2012). Disruption of these mechanisms during different developmental periods by neuronal injury can have long lasting functional consequences as we have observed in a model of pediatric hypoxia-induced ischemic injury. We showed that the early neural injury resulted in changes of several aspects of stimulus response properties. Response properties of VPm neurons from older rats were different than younger rats and some of the differences between sham and CA neurons persisted into these late periods. In particular, we found that average sensory evoked responses in CA-early rat neurons were elevated to levels similar to late sham and CA rats, while spontaneous activity level of CA-early neurons were intermediate to those of sham-early and both late groups. We found that the timing of initial whisker deflection responses in CA-early neurons were accelerated relative to sham-early neurons but still slower than either late group. Notably, we observed correlations between spontaneous activity and sensory evoked activity (timing and amplitude) in sham groups, but not in early or late CA groups. We also studied neural activity in the basal ganglia and MCx of sham and CA rats. We found that neurons in the EPN in the basal ganglia had lower firing rates yet had higher variability and higher levels of burst firing after injury. Correlated levels of power in LFPs between the EPN and the MCx were also disrupted with injury.

The present study extends the findings of previous related studies from our group examining neural dysfunction after pediatric CA-induced global brain hypoxia-ischemia (Shoykhet et al., 2012; Aravamuthan and Shoykhet, 2015). The prior studies revealed deficits in the average firing rates under spontaneous and stimulus-evoked conditions of thalamocortical neurons. Our study additionally characterizes the trial-to-trial variability of these neurons. Variability of neural activity can have significant impact on its ability to drive postsynaptic targets (Salinas and Sejnowksi, 2000). Slow-temporal correlations in neuronal spike trains result in Fano factors larger than 1 that increase with the counting window width (Middleton et al., 2003; Goris et al., 2014). In multiple sensory and motor areas of the brain, Fano factors universally decrease with the onset of sensory stimulation or the onset of motor planning and behavior (Churchland et al., 2006, 2010). Any differences in spontaneous or sensory evoked activity with neural injury may reflect changes in variability that compromise normal circuit function. We have also shown how spontaneous and sensory evoked activity, as well as stimulus response timing, is correlated with one another in normal and injured brain states. The present study also characterizes burst activity in the EPN in disease and normal states. What still remains unclear is how the parallel forms of dysfunction that we observed in sensory and motor systems may be interacting through development. Active whisking (motor) behavior can modulate how sensory information is processed (Fanselow and Nicholelis, 1999; Poulet and Petersen, 2008). Sensorimotor integration can occur at the level of the cortex (Ferezou et al., 2007), and behavior based on sensorimotor integration can exhibit deficits after ischemic injury (Hurwitz et al., 1991). Future studies are required to further understand that these injuries and age-related dysfunctions depend on one another.

Even though the functional differences we observe between neural activity in injured *vs*. sham rats in adulthood are fewer and smaller in magnitude than during early post-injury periods, the differences in activity we observe post-injury could have significant impact on sensory processing in the cortex. Altered thalamic input after CA likely has significant implications for experience-dependent plasticity in the developing cortex. In the rodent visual cortex, neonatal hypoxia-ischemia impairs ocular dominance plasticity following monocular deprivation (Failor et al., 2010). Concurrently, it alters the phenotype of inhibitory parvalbumin-expressing neurons and reduces overall activity levels. Thus, it has been suggested that neonatal hypoxia-ischemia impairs cortical plasticity by altering the development of inhibition in the visual cortex (Failor et al., 2010). Abnormal thalamic input, in turn, may contribute to dysfunctional cortical plasticity. In the rodent somatosensory system, altered sensory input during development disrupts maturation of inhibitory and excitatory cortical circuitry (Simons and Land, 1987; Shoykhet et al., 2005) and results in permanent behavioral deficits (Carvell and Simons, 1996). By analogy, abnormal thalamic input after CA may impact the functional maturation of cortical circuitry and result in long-lasting deficits. The central organization that we observed, reflected by the correlation of mean spontaneous activity and sensory-evoked activity magnitude and timing, likely has a significant impact on how spontaneous and sensory evoked activity of cortical neurons are correlated. This global change in population activity may affect the refinement of cortical circuits through the plasticity of recurrent synaptic activity during development. Further studies are required to assess functional changes in the somatosensory cortex in this ischemic injury model.

Ongoing spontaneous activity can engage short term plasticity of synaptic connections between neurons (Graham, 1977; Fujioka et al., 1994; Arbelaez et al., 1999; Reig et al., 2006; Choi et al., 2010) and may also be important for reinforcing stimulus-driven neural assemblies by engaging long term plasticity and homeostatic mechanisms (Myers and Yamaguchi, 1977; Pulsinelli and Brierley, 1979; Radovsky et al., 1997; Litwin-Kumar and Doiron, 2014). Subsets of neurons in the cortex that have higher spontaneous firing rates have specialized synaptic input composition that may increase their ability to exert strong excitatory influence on cortical activity (Myers and Yamaguchi, 1977; Pulsinelli and Brierley, 1979; Radovsky et al., 1997; Böttiger et al., 1998; Fink et al., 2004; Yassin et al., 2010). Spontaneously co-active sub networks of cortical neurons overlap with sensory evoked neuronal ensembles (Graham, 1977; Myers and Yamaguchi, 1977; MacLean et al., 2005). Spontaneous activity plays an important role in establishing and refining synaptic circuits in the brain during developmental periods. Based on our results we hypothesize that the covariation of spontaneous activity and stimulus-response amplitude and timing across a population of thalamic neurons may play an important role in the normal development of thalamocortical and intracortical synaptic circuits in sensory systems. The relationship between spontaneous and sensory-evoked activity, that CA neural activity lacks, may be important for reinforcing strong and organized thalamocortical sensory pathways during critical developmental periods. Additionally, burst firing may engage postsynaptic short-term plasticity mechanisms differently than the tonic or random Poisson firing at similar rates (Fortune and Rose, 2001; Middleton et al., 2011). All of these changes together may have important consequences on sensory processing and function of cortical neural networks in that it persist for much longer periods after early acute neural injury.

## Author Contributions

MS performed the experiments in this study. JWM performed the data analysis in this study. MS and JWM wrote the manuscript.

## Funding

JWM is funded by National Institutes of Health (NIH)/National Institute on Deafness and Other Communication Disorders (NIDCD) R03 DC012585, NIH/National Institute of General Medical Sciences (NIMGS) P30 GM103340 for JWM and National Institute of Neurological Disorders and Stroke (NINDS) K08 NS082362 for MS.

## Conflict of Interest Statement

The authors declare that the research was conducted in the absence of any commercial or financial relationships that could be construed as a potential conflict of interest.
